# Oculomotor differences in adults with and without probable developmental coordination disorder

**DOI:** 10.3389/fnhum.2024.1280585

**Published:** 2024-07-23

**Authors:** Emma Sumner, Elisabeth L. Hill

**Affiliations:** ^**1**^Department of Psychology and Human Development, Institute of Education (IOE), UCL’s Faculty of Education and Society, London, United Kingdom; ^2^Department of Psychology, City University of London, London, United Kingdom

**Keywords:** developmental coordination disorder, oculomotor, saccade, inhibition, eye tracking

## Abstract

Adults with Developmental Coordination Disorder (DCD), sometimes referred to as dyspraxia, experience difficulties in motor development and coordination, which impacts on all aspects of their daily lives. Surprisingly little is known about the mechanisms underlying the difficulties they experience in the motor domain. In childhood DCD, aspects of oculomotor control have been shown to be altered. The purpose of this study was to determine whether oculomotor differences are present in adults with and without probable DCD. Visual fixation stability, smooth pursuit, and pro-and anti-saccade performance were assessed in 21 adults (mean age 29 years) with probable DCD/dyspraxia (pDCD) and 21 typically-developing (TD) adults (mean age 21 years). Eye tracking technology revealed that oculomotor response preparation in the pro- and anti-saccade tasks was comparable across groups, as was pursuit gain in the slower of the two smooth pursuit tasks. However, adults with pDCD made significantly more saccades away from the fixation target than those without DCD and significantly more anti-saccade errors. Further, compared to TD adults, adults with pDCD demonstrated difficulties in maintaining engagement and had lower pursuit gain in the faster pursuit task. This suggests that adults with pDCD have problems with saccadic inhibition and maintaining attention on a visual target. Since this pattern of results has also been reported in children with DCD, oculomotor difficulties may be persistent for those with DCD across the lifespan. An awareness of the impact of atypical oculomotor control in activities of daily living across the lifespan would support clearer understanding of the causes and impacts of these difficulties for those with DCD.

## Introduction

Developmental coordination disorder (DCD), sometimes referred to as dyspraxia, has been estimated to affect between 5 and 6% of individuals (Blank et al., 2012). DCD is currently diagnosed using the Diagnostic and Statistical Manual (DSM-5) categorical framework, which identifies a significant delay in acquiring gross and/or fine motor skill as the primary characteristic (APA, 2013). Difficulties with motor coordination must also be seen to interfere with academic achievement and/or activities of daily living, and cannot be better explained by intellectual disability, visual impairment or a neurological condition affecting movement.

Theories about the underlying mechanisms of DCD are still to offer a definitive explanation about causality (Blank et al., 2019). A recent systematic review and meta-analysis of the literature highlighted consistent reporting of difficulties with executive functions (e.g., inhibition, working memory and attention which are supportive of movement control) and cognitive-motor integration [e.g., reduced patterns of activation when considering functional Magnetic Resonance Imaging (fMRI) data during tasks that required different aspects of action preparation and attention during motor performance] in children with DCD (Subara-Zukic et al., 2022). Taken together, the authors suggested that DCD may be a disorder of motor-cognitive function. Studying oculomotor (eye movement) function using tasks that tap into executive function (e.g., planning, inhibition or shifting of eye movements) and tracking targets (visual-motor integration) is one way to further explore aspects of both motor and cognitive control (Maron et al., 2021).Notably little data is available to indicate whether these difficulties persist into adulthood in those with DCD. However, such a finding has important research, clinical and educational implications, in terms of developing our theoretical understanding of DCD and targeting support.

The visual system enables us to process information in the world that we can then act upon. By focusing the eyes, *fixation* enables an individual to determine what and where an object is (Krauzlis et al., 2017). Eye movements (*saccades*) are made to redirect the fovea and centre an object on this region of the retina, thus making the object clearer (Karatekin, 2007). These saccades may be reflexive (i.e., moving the eyes to a new object that has appeared in the visual system) or volitional (i.e., involving high-level control, such as using spatial memory to search for an object, or inhibition to avoid distractions). Of note, there is considerable overlap of the networks involved in oculomotor control, cognitive control (e.g., attention, planning, inhibition) and motor coordination (e.g., fronto-cerebellar and fronto-parietal regions; Doron et al., 2010; Kheradmand and Zee, 2011). Oculomotor paradigms that explore inhibitory control include asking individuals to sustain fixation, to make a saccade to an object (pro-saccade), or to inhibit a reflexive eye movement to an object appearing in the periphery (e.g., using an anti-saccade task, explained later). Another assessment of oculomotor control is *smooth pursuit*, which involves maintaining an object on the fovea, thus the eye moves with the object, such as when catching a ball or tracking a moving object (Karatekin, 2007). Precise coupling between oculomotor and limb kinematics is important for motor planning and coordination. Poor pursuit has been associated with eye-hand coordination difficulties, such as catching a ball (Glazebrook et al., 2009), and motor planning (e.g., when reaching for an object) is supported by attention and visual-motor integration (Gilbert and Li, 2013). Thus, mastery of accurate oculomotor control is one important aspect for the acquisition and execution of skilled behaviors (e.g., completing fine motor tasks, locomotor control, navigating the environment).

Atypical visuomotor function has been widely documented in children with DCD, with studies revealing difficulties in orienting attention (using COVAT (Covert Orienting of Visuospatial Attention Tasks) e.g., Wilson et al., 1997; Chen et al., 2012), and delays in attentional disengagement and motor initiation in hand-eye coordination tasks (e.g., Wilmut et al., 2007; Wilson et al., 2017). Given the relationship between the visual and motor systems, it has been argued that it is possible that atypicalities in oculomotor function will have consequences for broader fine and gross motor skill difficulties (Maron et al., 2021). Yet, research utilising oculomotor paradigms in individuals with DCD remains scarce.

One study to explore saccade performance in a small sample of adolescents with dyspraxia (reported as being diagnosed with DCD as per the DSM-5 criteria; *n* = 7) revealed a mixed pattern across different saccade tasks with varying demands (e.g., making visually-guided saccades, memory-guided saccades, delayed saccades, and anti-saccades; Gaymard et al., 2017). Characteristics of those with dyspraxia compared to age-matched controls revealed increased variability of saccade amplitudes in the visually guided task, decreased velocities of non-visually guided saccades, in addition to higher error rates on an anti-saccade task. The authors concluded that the findings may reflect impaired connectivity between frontal and cerebellar oculomotor structures in a dyspraxic sample. However, since their small sample comprised adolescents with dyspraxia all co-occurring with other conditions (including autism, schizophrenia and reading difficulties), further work is needed to understand the specificity of this conclusion.

Another study to examine oculomotor function focused on primary schoolaged children with a diagnosis of DCD (*n* = 23). Sumner et al. (2018) found that children with DCD were comparable to typically-developing peers in their ability to prepare and direct an eye movement (i.e., saccade latency and accuracy was comparable in a visually-guided saccade task, often referred as a ‘prosaccade’ task). However, children with DCD presented with difficulties maintaining engagement in fixation and smooth pursuit tasks when compared to a control group and, echoing the findings of Gaymard et al. (2017) made more errors on an anti-saccade. The finding of difficulties with pursuing a moving target supports earlier findings showing that children with DCD had lower pursuit gain than their peers and made more saccades away from the target (Langaas et al., 1997). Sumner et al. (2018) argued that children with DCD have pronounced difficulty exercising inhibitory control (i.e., suppressing intrusive saccades); a finding that implicates the fronto-parietal circuit (Miller et al., 2005). These findings are also supported by Gonzalez et al. (2016) who found that children with DCD made more saccades away from a target and had difficulty inhibiting saccades.

To the best of our knowledge, research on oculomotor function in DCD has not been extended to adult populations. This is despite the increasing recognition that motor difficulties persist into and throughout adulthood for some individuals with DCD (e.g., Tal-Saban and Kirby, 2018). Being able to further characterize oculomotor function in DCD by extending this to adults, is an important step in understanding whether a unique oculomotor profile may present (and be persistent) in those experiencing motor difficulties. Thus, the aim of the current study was to extend the use of the oculomotor paradigm cited in Sumner et al. (2018) to an adult population, asking: do adults with DCD present with oculomotor challenges in comparison to adults that do not have a diagnosis of DCD? Given the existing findings that highlight executive functioning difficulties as a prominent feature of DCD at all ages (Meachon et al., 2022), a brief measure of self-reported attention difficulties was taken to characterize the study sample in this respect, along with self-reported diagnosis of any conditions other than DCD. More detailed consideration of attention deficits and executive functioning of various forms was not possible within the scope of the current study. A body of research has shown difficulties with inhibition in children and adults with DCD using experimental tasks (e.g., Bernardi et al., 2016; He et al., 2018). Thus, based on existing research from both eye tracking and experimental studies, we predicted difficulties in saccadic inhibition but not saccade preparation in adults with DCD compared to typically-developing adults. Such difficulties were expected to be observed in a fixation and smooth pursuit task, which require inhibiting saccades and maintaining fixation on the target (more saccades were predicted for the fixation task, and less time in pursuit); as well as during an anti-saccade task that requires facilitation of saccades away from a target (more errors were predicted in this task).

## Materials and methods

### Participants

Two groups of participants were recruited: 21 adults who reported a diagnosis of DCD/dyspraxia (aged 21–46, 10 male) and 21 typically-developing adults that did not report a motor difficulty (aged 18–32, 5 male). Participants were recruited via a research participant scheme in a university, by contacting university disability services in England and through a targeted approach on social media.

Adults with DCD/dyspraxia completed a screening questionnaire designed by the research team in which they confirmed their date of birth, ethnicity and any formal developmental, physical or medical diagnoses that they had received from birth to the present day. Five participants reported having a co-occurring diagnosis of dyslexia. No other developmental or medical diagnoses were reported. Since a comprehensive diagnostic assessment could not be undertaken, henceforth this group is described as ‘probable DCD’ (pDCD). Fifteen participants (71.4%) self-identified as White British, two as White Other (9.6%), two as Indian Asian (9.6%), one as Black (4.7%) and one as Mixed Heritage (4.7%). Two participants wore contact lenses during testing.

Typically developing adults in the comparison group also completed the screening questionnaire. No adult in this group reported any diagnosis of any kind (development, physical or medical). Sixteen participants self-identified as White British (76.1% of the sample), two as Black (9.6%), two as Mixed Heritage (9.6%) and one as Indian Asian (4.7%). One participant wore contact lenses during testing.

### Measures

#### Adult ADHD self-report scale

The Adult ADHD Self Report Scale v1.1 (ASRS) is a checklist that can be used as a screening tool for ADHD in the general population (Kessler et al., 2005). The checklist consists of 18 questions (part A and B) that ask the individual to rate the frequency of different clinical manifestations based on the DSM-5 criteria for ADHD, with responses ranging from ‘Never’ to ‘Very Often’. If the participant scores 4 or more in Part A they are considered at high risk of adult ADHD. Part B provides additional cues. Kessler et al. (2005) reported a sensitivity of 68.7% and a specificity of 99.5% for identification in a population-based sample. The overall sum of scores (0–18) was used for analyses.

#### Oculomotor battery

The set-up and tasks were administered in the same way as in Sumner et al. (2018).^1^ Eye movements were recorded at a sampling rate of 1,000 Hz using the Eyelink 1,000 eye tracker (SR-research). The camera was positioned using the desktop mount placed below the computer screen and a chin rest was used to keep the head stable. The experiment was implemented using Experiment Builder and analyzed using Data Viewer (both SR Research software).

The oculomotor battery comprised four tasks, with participants seated directly in front of the computer monitor at a viewing distance of approximately 80 cm. In each task, a trial started with a fixation cross in the centre of the screen, followed by the stimulus/target consisting of a red circle presented against a black background on the computer screen with 1,024 × 786 screen resolution. The red circle measured 0.65° × 0.65° visual angle. Written (on-screen) and verbal instructions were provided. Breaks were included between tasks, if necessary.

(i) In the visual *fixation* task, participants were instructed to maintain their gaze on the target shown in the centre of the screen, until it disappeared. The task lasted for 30 s and began after a drift correct procedure.

(ii) Two *smooth pursuit* tasks, at differing speeds, were administered. Participants were required to follow the target (i.e., keep their eyes on the target) which had a horizontal sinusoidal motion, moving at 0.2 Hz (slow trial) and then at 0.5 Hz (fast trial). Each trial lasted 20s and was preceded by a drift correct procedure. The target traveled 8.5°/s in the slow trial and 21.5°/s in the fast trial.

Both the (iii) prosaccade and (iv) anti-saccade tasks used a ‘step’ procedure, meaning that the cue disappeared at the same time as the peripheral target appeared. Each of the 24 trials per task was preceded by a drift correct procedure which then moved on to display the central fixation target. The central target was displayed for 1,000 ms before moving either left or right (on the horizontal meridian 6.25°). The direction of the step was randomized in both tasks and the target was displayed in the new location for 1,000 ms. For the *prosaccade* task (sometimes referred to as a ‘visually-guided’ saccade task), participants were instructed to look at the central fixation point and then move their eyes as quickly as possible to the target when it moved from the central point. For the *anti-saccade* task, the procedure remained the same, but participants were instructed to ignore the target when it moved from the centre of the screen and to look as quickly as possible in the opposite direction. The instructions were explained and then verified with the participant.

### Procedure

Ethical approval was obtained from Goldsmiths, University of London, research ethics committee. Participants provided informed written consent prior to attending a research visit. The questionnaire and oculomotor battery were completed in one session, lasting no longer than 45 min. Participants were seen individually in a quiet room, which was dimly lit for the eye tracking tasks. Participants were first introduced to the eye-tracking set up. They took part in a 5-point calibration exercise at the beginning of the eye-tracking session, which was repeated as required. The oculomotor test battery was undertaken in a fixed order (as presented above). All participants completed the four oculomotor tasks.

### Eye tracking data analysis

#### Fixation task

Four measures were taken to assess active engagement on the target (e.g., fixation ‘stability’): Time on Target (within 1° visual angle, represented as a %); Number of saccades during the 30s task; Average fixation duration; Weighted distance of saccades away from the target.

#### Smooth pursuit

Eye movements in these trials were quantified using customized software written in LabView. Four key metrics of smooth pursuit performance are presented: Number of qualifying pursuit segments, Sum of durations of qualifying pursuit segments (i.e., time spent in pursuit), Weighted average velocity gain, Weighted average root-mean-square-error (RMSE). Pursuit segments were extracted and the duration of each segment was determined using the online parsing decisions made by the eye tracking software. To identify a pursuit segment (e.g., when the eye was moving/following the target), first samples were recorded as being in a saccade if the sample velocity exceeds 30°/s, or acceleration exceeds 8,000°/s^2^. All samples that were not classified as being part of a saccade (or a blink – which includes periods of ‘tracking loss’) were considered as being in a potential pursuit segment. The number of these identified pursuit segments were then calculated per task and individual, as well as identifying the time in pursuit (summing the time of all identified pursuit segments). As set out in Sumner et al. (2018), pursuit gain calculated the ratio of the eye velocity to target velocity (i.e., the average of eye velocity divided by target velocity for each pursuit segment). RMSE was calculated as the square root of the average of eye position (in degrees of visual angle) subtracted from target position (in degrees of visual angle) squared. Any pursuit segments with velocity gain values below 0.5 or above 1.5 were removed prior to analysis, as were pursuit segments less than 100 ms in duration, and with RMSE values of above 2. For each target speed, the average Gain and RMSE measures were weighted by the duration of pursuit segments.

#### Prosaccade and anti-saccade

For trials to be considered ‘valid’ for analysis, participants had to be fixating on the central fixation point at target onset and the start time of the first saccade had to be >80 ms (i.e., not anticipatory). Two measures were calculated for both tasks: saccade latency (ms) and percentage of direction errors. In addition, accuracy (in terms of amplitude – i.e., how close the eye movement was to the target - reported in degrees of visual angle), was also measured in the prosaccade task. This calculation is based on the screen pixel co-ordinates of the gaze data, using parameters for screen height, width, distance and pixel resolution.

### Statistical analysis

Normality of data were checked considering the histograms, Q-Q plots and the Shapiro–Wilk’s W test (*p* > .05 indicating normally distributed data). Parametric tests (ANOVAs) were conducted to explore group differences in the eye tracking measures on normally distributed data, while non-parametric equivalents (Mann Whitney) were conducted for non-normally distributed data. The sample size reported here is similar to that reported in Sumner et al. (2018), *n* = 24 DCD children. As adults with pDCD differed from the comparison group in terms of age and the ASRS score, Bivariate correlations (using the whole sample) were conducted with the eye tracking measures to determine whether these variables should be controlled for in subsequent analyses.

## Results

Table 1 presents the age and ASRS scores for the two groups of participants, as well as the group comparison statistics. The pDCD group was significantly older than the TD group. Although none had reported a diagnosis, a higher proportion of adults with pDCD (*n* = 14, 66.6%) met the criteria for in-depth ADHD evaluation according to the ASRS checklist (e.g., scoring ≥4 on Part A), compared to just one participant in the TD group (4.7%). Considering the overall ASRS score, adults with pDCD scored significantly higher than the comparison group; although of note, a range of scores are observed for the pDCD group.

**Table 1:** Participant characteristics.

	TD (*n* = 21)	DCD (*n* = 21)	Group comparison
Age in years Mean (SD) Range	21.81 (4.72) 18–32	29.86 (7.91) 21–46	*t* (4,40) = 8.77, *p* < 0.001
ASRS overall score Mean (SD) Range	4.57 (1.32) 3–7	8.42 (4.62) 1–18	*U* = 68.00, *p* < 0.001

ASRS, ADHD Self-Report Scale, overall score maximum of 18.

Bivariate correlations were conducted using age and the ASRS overall score along with the subsequent eye tracking measures reported below. Age and ASRS were found to be positively correlated (*r* = 0.59, *p* < .001). Bonferroni adjustment of the level of significance was calculated based on the 16 eye tracking measures used for the correlational analysis (0.05/16 = *p* < .003). Based on a significance level < .003, neither age nor the ASRS score were found to correlate with any of the eye tracking measures. Due to the lack of significant correlations and the distribution of data for some of the measures which meant that non-parametric tests needed to be conducted, age and ASRS have not been included as covariates in the subsequent analyses.

### Fixation

The fixation findings are shown in Table 2. Mann Whitney U-tests were conducted due to the data not being normally distributed. Significant group differences were reported for the number of saccades, average fixation duration and time on target; with adults with pDCD making more saccades during the fixation task, fixating less and spending less time on the target than the TD group. No group differences were observed for the distance the eyes moved from the target.

**Table 2:** Group comparisons on the fixation measures.

	TD (*n* = 21)	DCD (*n* = 21)	Group comparison
Number of saccades Mean (SD) Range	16.43 (8.74) 5–37	32.62 (21.75) 9–79	U = 128.50, *p* = .02
Average fixation duration (s) Mean (SD) Range	2.20 (1.26) 0.73–4.97	1.28 (0.83) 0.29–2.92	U = 126.00,*p* = .01
Time on target (s) Mean (SD) Range	27.46 (17.12) 17.12–29.96	25.13 (5.47) 11.06–29.69	U = 139.00, *p* = .04
Weighted distance from target Mean (SD) Range	13.61 (10.84) 2.55–46.19	27.53 (51.05) 2.89–185.67	U = 206.00, *p* = .72

s, seconds.

### Smooth pursuit

The two trials (slow and fast) for the smooth pursuit tasks are reported in Table 3. Two-way repeated measures ANOVAs [2 (group: TD, pDCD) x 2 (trial: slow, fast)] were conducted for each of the four measures. Significant Group x Trial interactions were identified in the analysis of duration of time spent in pursuit, *F*(1,40) = 8.39, *p* = .006, *n^2^_p_* = 0.17, and pursuit gain, F(1,40) = 4.69, *p* =.03, *n^2^_p_* = 0.11. For pursuit duration, there was a significant effect of group, *F*(1,40) = 6.19, *p* = .02, *n^2^_p_* = 0.13, and the same for the gain value, *F*(1,40) = 3.80, *p* = .05, *n^2^_p_* = 0.09. For both duration and gain measures, the interaction effects revealed that adults with pDCD showed poorer performance than the TD group in the faster trial (less time in pursuit: *p* = .006, and lower gain: *p* = .03).

**Table 3:** Group comparisons on the smooth pursuit tasks.

	TD (*n* = 21)	DCD (*n* = 21)
*Slow*		
Number of segments Mean (SD) Range	21.62 (7.32) 12–36	25.29 (6.08) 13–43
Duration (s) Mean (SD) Range	15.53 (2.41) 9.21–18.22	14.37 (2.66) 8.56–17.66
Weighted gain Mean (SD) Range	1.03 (0.05) 0.96–1.16	1.00 (0.08) 0.84–1.14
Weighted RMSE Mean (SD) Range	0.67 (0.31) 0.31–1.45	0.64 (0.25) 0.26–1.11
*Fast*		
Number of segments Mean (SD) Range	34.48 (8.54) 22–52	31.57 (13.84) 10–60
Duration (s) Mean (SD) Range	14.15 (3.17) 8.57–18.21	10.69 (4.49) 2.32–16.33
Weighted gain Mean (SD) Range	0.95 (0.09) 0.78–1.21	0.88 (0.11) 0.64–1.03
Weighted RMSE Mean (SD) Range	1.04 (0.19) 0.68–1.39	1.02 (0.14) 0.79–1.30

The Group x Trial interactions were not significant for the measures of pursuit segments, *F*(1,40) = 3.42, *p* = .07, *n^2^_p_* = 0.08, or RMSE, *F*(1,40) = 0.82, *p* = .37, *n^2^_p_* = 0.02; nor were there any significant overall group differences, *F*(1,40) = 0.03, *p* = .87, *n^2^_p_* = 0.00 and *F*(1,40) = 0.02, *p* = .89, *n^2^_p_* = 0.00, respectively.

### Pro- and anti-saccade

Table 4 reports the pro- and anti-saccade findings. Analyses were based on ‘valid’ trials, as discussed in the Methods section. For the prosaccade task, the mean (SD) and range of valid trials out of 24 per group were: pDCD, *M* = 20.04 (2.87), 14–24; TD, *M* = 21.38 (2.99), 12–24; and for the antisaccade task also out of 24 pDCD, *M* = 21.19 (2.78), 15–24; TD, *M* = 22.14 (2.10), 16–24. The two groups performed similarly on prosaccade latency, but the TD group had significantly shorter latencies in the anti-saccade task than adults with pDCD. No group difference was found for prosaccade amplitude. A significant group difference was found for anti-saccade error rate, with adults with pDCD making more errors.

**Table 4:** Group comparisons on the prosaccade and antisaccade tasks.

	TD (*n* = 21)	DCD (*n* = 21)	Group comparison
*Latency*			
Prosaccade (ms) Mean (SD) Range	159.24 (12.54) 139.79–180.05	164.60 (34.04) 122.73–271.76	*t*(40) = 0.67, *p* = .50
Antisaccade (ms) Mean (SD) Range	232.10 (29.87) 183.08–285.29	268.41(58.56)^a^ 87.50–355.75	*t*(39) = 2.52, *p* = .02
*Amplitude*			
Prosaccade Mean (SD) Range	6.87 (0.51) 5.48–7.73	6.79 (0.43) 5.83–7.56	*t*(40) = −0.53, *p* = .59
*Error rate*			
Antisaccade (%) Mean (SD) Range	15.19 (12.67) 0–45	41.85 (29.17) 9–100	*U* = 84.50, *p* = .001

^a^1 missing data point due to timing recording error; amplitude in degrees, the target moved 6.25° to the left or right.

## Discussion

The aim of the present study was to consider oculomotor function in adults with probable DCD. This brief report presents the first analysis of the oculomotor profile of adults with pDCD through the administration of a battery of tasks assessing eye movements (e.g., fixations, saccades and smooth pursuit) and high-level (e.g., inhibitory control) cognitive control processes involved in oculomotor control in adults with and without pDCD. Using the same tasks as reported in Sumner et al. (2018) further allows for an indirect comparison to the profile of primary schoolaged children with DCD who were the focus of that study. Encouragingly, the present findings revealed that the underlying mechanisms for preparing and executing saccades (*pro-saccade* task: initiating an eye movement to a target and arriving at that target) and engaging in pursuit of a slow moving target were found to be comparable between adults with and without pDCD. This finding means that low level oculomotor processes could be considered to be intact in adults with pDCD.

However, notable difficulties were observed between adults with and without pDCD in the tasks that required fixation to one position on the screen and suppressing eye movements (fixation task) or suppressing a reflexive saccade (anti-saccade task). In both of these tasks, adults with pDCD were shown to have difficulty with saccadic inhibition. It could also be argued that the finding of adults with pDCD spending less time pursuing the target in a fast smooth pursuit trial and lower gain compared to the typically developing group points toward a higher number of saccades (eye movements) being made. Together these findings suggest a difficulty with exerting top-down cognitive control, which may be attributed to interference with the fronto-parietal networks and under-development of a control network important for saccade inhibition (Gonzalez et al., 2016). Parallels can be drawn to both research findings and the self-evaluative descriptions of those with DCD identifying challenges for many across a range of executive function tasks (including inhibition) both in the lab and in daily life (e.g., Tal Saban et al., 2014; Bernardi et al., 2018; Scott-Roberts and Purcell, 2018; Sartori et al., 2020; Lachambre et al., 2021; Abdollahipour et al., 2023; Fogel et al., 2023). Moreover, emerging data suggests altered neural structure, structural and functional connectivity and neurophysiological activity across multiple brain regions in people with DCD, within and across sensori-motor and prefrontal regions (e.g., Wilson et al., 2017; Rinat et al., 2020; Subara-Zukic et al., 2022). While such differences are also apparent in people with ADHD, there is emerging evidence of shared and distinct neural correlates in each diagnostic group (Kangarani-Farahani et al., 2022).

The findings of low-level oculomotor processes being intact (e.g., preparing and executing saccades), but disturbances in saccadic inhibition shown in adults with pDCD echo those from a child sample (Sumner et al., 2018). Interestingly, Sumner et al. found group differences between children with and without a diagnosis of DCD in the measure of pursuit duration for both slow and fast pursuit trials, whereas adults in the current sample appeared only to have difficulty with the faster trial. Adults with pDCD demonstrated compromised (slow) processing of online feedback when responding to a faster paced target, as the task requires online prediction of the target position. This may suggest a slowed process of natural development over time or the development of an alternative approach to slow pursuit in those with DCD. Considered alongside some evidence of brain based changes in children with DCD emerging through targeted intervention (Izadi-Najafabadi and Zwicker, 2021), and given the importance of oculomotor control for effectively completing activities of daily living, this possibility warrants further investigation.

Difficulties with attention and saccadic inhibition, evidenced both in a child sample (Sumner et al., 2018) and the current adult sample, may be one explanation for the challenges that individuals with DCD experience in the acquisition of skilled behaviors that often rely on visual attention. Al-Yahya et al. (2023) highlight that interventions targeting motor skill training for individuals with DCD often demonstrate variable performance and suggest that we need to consider the underlying mechanisms of DCD to further intervention approaches. Inhibitory control supports the execution of day-to-day tasks and these oculomotor control mechanisms support the allocation of visual attention (e.g., directing saccades to stimuli; Gonzalez et al., 2016). Often movement requires rapid processing of visual information. For example, Wood et al. (2017) reported improvements in ball catching for children with DCD following training of saccadic and fixation behaviors. This finding supports a link between oculomotor control and guided motor performance and may be an area for future intervention research.

While the current study is a preliminary first step to explore oculomotor performance in adults with pDCD, limitations can be acknowledged. It was not possible to conduct an assessment of motor skill to confirm the diagnosis of DCD and we relied on self-report of a diagnosis. Knowledge of when participants’ received their diagnosis of DCD may have been useful to consider, as it could be that early diagnosed individuals may have received support and developed their skills in different ways to those with later diagnoses. Five pDCD participants reported a dyslexia diagnosis and while these two conditions are known to co-occur it was not possible to consider the implications of this within the study although this may be of interest in the future given reports of subtle inhibition difficulties noted in oculomotor tasks completed by adults with dyslexia (Wilcockson et al., 2019). Further, the increased prevalence of attention difficulties (measured by the ASRS) in the pDCD group may raise questions about potential interactions with attention difficulties and the impact on the findings. However, of note, data reported by Tal Saban et al. (2014) suggests that in a sample of 284 young adults with DCD and those categorized as having borderline DCD, reported problems with executive function remain consistent with or without attention difficulties. It was not possible to control for attention difficulties in the current analysis due to statistical restrictions. However, this measure was not found to correlate with the eye tracking measures and participants did not report a clinical diagnosis of ADHD. Co-occurrence of DCD and ADHD is reportedly high (Blank et al., 2019) and studies have shown a similar pattern of saccadic inhibition difficulties, but no problems with pursuit of a target in individuals with ADHD (Maron et al., 2021). Further research is warranted to understand the overlap between DCD and ADHD in this respect and the impact on cognitive control and motor skill acquisition. Finally, we make indirect cross-sectional comparisons between the findings from existing child data (Sumner et al., 2018) and the current adult data. While the child and adult data that are compared in our evaluation are not drawn from a single study, we argue that they are directly comparable given that they completed identical oculomotor tasks in the same lab. Future research in this area should seek to examine oculomotor performance longitudinally in individuals with DCD, explore sex differences of oculomotor control in DCD, as well as relating oculomotor performance to measures of fine and gross motor skill performance.

To conclude, the current findings provide evidence of oculomotor disturbances in adults with pDCD particularly in relation to eye movements that engage top-down cognitive control processes (i.e., saccadic inhibition and maintaining gaze to a central fixation point). Given the criticality of inhibitory control for managing day-to-day tasks and the consequent impact on activities of daily living, academic and employment outcomes, as well as broader factors such as mental health and wellbeing, these findings highlight the need for recognition of the impact of DCD and the ongoing challenges in adulthood. They are also likely to impact on skill acquisition that continues through the lifespan. Further research in this area in relation to both DCD and other neurodevelopmental disorders could demonstrate potential biomarkers of DCD for research and clinical practice, aiding clearer understanding and identification of DCD in adulthood and approaches for intervention.

## Data availability statement

The raw data supporting the conclusions of this article will be made available by the authors, without undue reservation.

## Ethics statement

The studies involving humans were approved by the Goldsmiths, University of London, ethics review committee. The studies were conducted in accordance with the local legislation and institutional requirements. The participants provided their written informed consent to participate in this study. Written informed consent was obtained from the individual(s) for the publication of any potentially identifiable images or data included in this article.

## Author contributions

ES: Conceptualization, Data curation, Formal analysis, Investigation, Methodology, Project administration, Writing – original draft, Writing – review & editing. EH: Funding acquisition, Writing – original draft, Writing – review & editing.

## Footnotes

^1^Note that Figures showing the experimental set up and the pro- and anti-saccade tasks are as depicted in Sumner et al. (2021).
